# A Two-Phase Space Resection Model for Accurate Topographic Reconstruction from Lunar Imagery with PushbroomScanners

**DOI:** 10.3390/s16040507

**Published:** 2016-04-11

**Authors:** Xuemiao Xu, Huaidong Zhang, Guoqiang Han, Kin Chung Kwan, Wai-Man Pang, Jiaming Fang, Gansen Zhao

**Affiliations:** 1 School of Computer Science and Engineering, South China University of Technology, Guangzhou Higher Education Mega Centre, Guangzhou 510006, China; z.huaidong@mail.scut.edu.cn (H.Z.); chanme0923@gmail.com (J.F.); 2 School of Computing and Information Sciences, Caritas Institute of Higher Education, Tseung Kwan O, N.T., Hong Kong 999077, China; kckwan@cse.cuhk.edu.hk (K.C.K.); wmpang@ieee.org (W.-M.P.); 3 School of Computer Science, South China Normal University, Guangzhou 510631, China; gzhao@scnu.edu.cn

**Keywords:** space resection, recovery of exterior orientation parameters, lunar topographic reconstruction, push broom scanners, lunar elevation measurements

## Abstract

Exterior orientation parameters’ (EOP) estimation using space resection plays an important role in topographic reconstruction for push broom scanners. However, existing models of space resection are highly sensitive to errors in data. Unfortunately, for lunar imagery, the altitude data at the ground control points (GCPs) for space resection are error-prone. Thus, existing models fail to produce reliable EOPs. Motivated by a finding that for push broom scanners, angular rotations of EOPs can be estimated independent of the altitude data and only involving the geographic data at the GCPs, which are already provided, hence, we divide the modeling of space resection into two phases. Firstly, we estimate the angular rotations based on the reliable geographic data using our proposed mathematical model. Then, with the accurate angular rotations, the collinear equations for space resection are simplified into a linear problem, and the global optimal solution for the spatial position of EOPs can always be achieved. Moreover, a certainty term is integrated to penalize the unreliable altitude data for increasing the error tolerance. Experimental results evidence that our model can obtain more accurate EOPs and topographic maps not only for the simulated data, but also for the real data from Chang’E-1, compared to the existing space resection model.

## 1. Introduction

Acquiring lunar topographic data is an important mission for lunar exploration, and the push broom scanner is one of the critical optical devices carried by orbiters for acquiring stereo lunar CCD images (e.g., Chang’E-1 [1] and Chang’E-2 [2]). To reconstruct the lunar topography based on these stereo CCD images, a rigorous bundle adjustment framework [2,3,4] is usually adopted, which is greatly influenced by the accuracy of the exterior orientation parameters (EOPs) of the push broom scanner. Unfortunately, the EOP information is unknown during the acquisition of stereo lunar CCD images. Therefore, how to obtain the accurate EOP information of the push broom scanner is the key issue for lunar topographic reconstruction.

**Figure 1:**
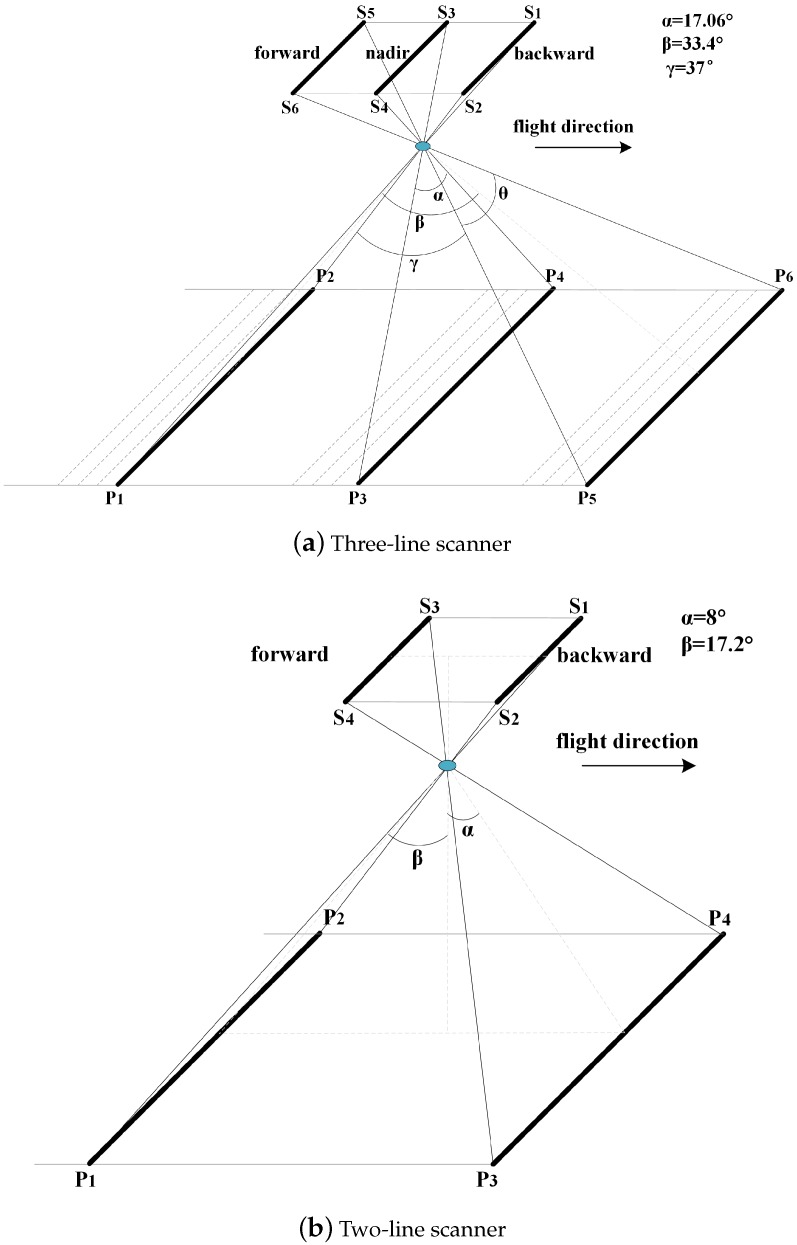
The push boom scanners equipped on (**a**) Chang’E-1 and (**b**) Chang’E-2.

The push broom scanner is popular due to its simple linear scanning mechanism (Figure 1), while it also complicates the recovery of its EOPs as the scanner captures the CCD images during flight. This implies that its EOPs would be varied at each instant of time for each scanline. Classical frame imagery methods are not applicable to this special situation. In order to estimate the EOPs for each scanline, *space resection* [5] is a basic and critical model which determines the six parameters in EOPs (three coordinates of *spatial position* and three elements of *angular rotation*) based on the *ground control points* (GCPs). Here, a GCP refers to a location on the lunar surface and can be presented by its 3D coordinate (usually referred to as longitude, latitude and altitude). The space resection method has been proven to be effective when the data of the GCPs are error-free.

**Figure 2:**
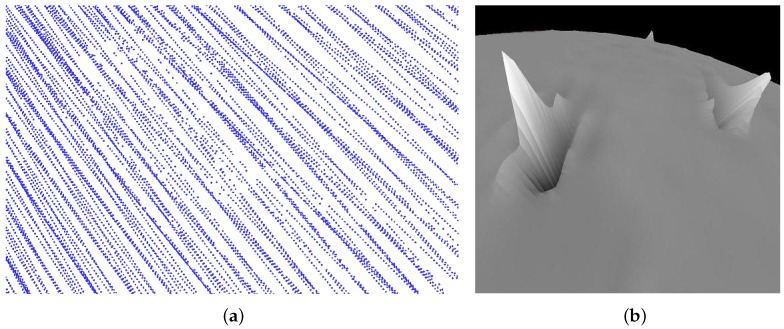
Laser altimeter measurements (LAM) data from Chang’E-1. (**a**) The non-uniform distribution of LAM data; (**b**) the noises in LAM data cause the sudden peaks in the landscape.

However, since existing modeling of space resection [5] involves the linearization of the collinearity condition and the use of an iterative process to determine the solution using the nonlinear programming approach, it cannot guarantee a global optimal solution and is highly sensitive to the initial guess of the unknown parameters. This problem becomes even serious when the GCPs contain errors. Fortunately, the *geographic data* (longitude and latitude) of the first and last pixels of each scanline in CCD images are directly captured by the orbiters of the Chang’E series and provided in the PDS (Planetary Data System) files. These geographic data should have a very low level of error. Thus, in our work, we take the ground points on the lunar surface corresponding to these pixels as the GCPs. Note that the longitude and latitude data are insufficient to obtain the accurate coordinates of these GCPs. The altitude data of GCPs are also required. However, the altitude data are unavailable and are usually required to be interpolated from the sparse laser altimeter measurements (LAM) data. Even worse, the existing LAM data [6] usually are distributed unevenly (Figure 2), which will inevitably introduce errors into the interpolated altitude data and further decrease the accuracy of the estimated EOPs. In this regard, how to increase the error tolerance for the modeling of space resection is the key concern to achieve an accurate EOP recovery.

To tackle the above problem, we study the mechanism of the push broom scanner (Figure 1) in detail and find that the estimation of angular rotation parameters of EOPs can be computed independent of the altitude data and only involving the geographic data at the GCPs. This motivates us to improve the estimation of EOPs in a divide-and-conquer way. By dividing the estimation of EOPs into two phases, the accurate *angular rotation* parameters of EOPs can be first determined based on the error-free geographic data at the GCPs using our proposed mathematical model. In the second phase, based on the accurate angular rotations, the collinear equations for space resection can be simplified into a linear optimization problem, which guarantees that the globally optimal *spatial position* of EOPs can be always achieved. This also implies that the choice of initial values for unknown parameters has a minor effect on the final solution. On the other hand, since the lunar surface is pitted with craters while LAM data were sampled non-uniformly, the interpolated altitude data at the GCPs may contain large errors. To penalize the unreliable altitude data, we propose a new certainty metric for the interpolated altitude data and further integrate this certainty into the optimization process. This enables us to achieve robust estimation of spatial position even if some of the interpolated altitude data at the GCPs suffer from large errors.

To evaluate our method, we carry out experiments on two data sources, including the simulated data and real data from Chang’E-1. The simulated data are used as the ground truth data in testing our algorithms, since the ground truth is not available. For the experiment on the simulated data, we design multiple models to generate the simulated lunar surface, simulated orbiter trajectory, simulated LAM data and simulated stereo images. Based on the simulated data, two kinds of validations are conducted. One is to validate the improvement of the EOPs’ estimation by our model compared to the existing model of space resection. The other is to validate the quality in generating the topographic maps using our estimated EOPs *versus* those by the existing model of space resection. This experiment also demonstrated that the topographic maps are easily affected by the error of EOPs. For the experiment on the real data, we first recover the trajectory using our model of space resection and then utilize image matching and forward intersection [7] to reconstruct the 3D coordinates for the pixels on CCD images. Experimental results show that based on our estimated EOPs, our method can obtain more reasonable altitude maps for CCD images, compared to the existing model of space resection.

## 2. Related Work

Currently, push broom imagery [8] is a fundamental technology for airborne digital sensors to collect high-resolution stereo images. It has been successfully applied to satellite photogrammetry for Earth observation (e.g., Digital Photogrammetric Assembly (PDA) [9], Modular Opto-electronic Multi-spectral Stereo Scanner (MOMS-02/D2) [10], SPOT5 [11], QuickBird [12,13], IKONOS [13,14,15], WorldView and GeoEye), Mars observation (e.g., High Resolution Stereo Colour Imager (HRSC) [16]) and Lunar observation (e.g., CE-1 [1,17,18], CE-2 [2,3]). Besides, the push broom scanner has also been widely exploited in aerial photogrammetry, such as Airborne Digital Sensor (ADS40) [13,19], StarImager [20,21,22,23] and Digital Airborne Scanner (3-DAS-1) [23]. In order to recover the EOPs of the push broom scanners and to further reconstruct the planet topographic maps accurately, many works have been explored and can be generally classified into three categories.

The initial solution was to model the trajectory of the scanner by piecewise polynomial models, since the orbiter that carried the scanner was supposed to move stably and smoothly in neighboring scanlines. For example, Wang [24] adopted an equivalent frame photo bundle adjustment method, which discretized the image into multiple parts and assumed each part had the same set of extrinsic parameters. However, this model heavily relied on the initial values of EOPs. Moreover, it accumulates error in the iterations and, finally, leads to a shift from the true trajectory.

Secondly, GPS/IMU-aided processing of push broom imagery has been another popular way for aerial photogrammetry. However, due to the inevitable systematic errors from the GPS/IMU, directly using the EOPs acquired by the onboard GPS/IMU may introduce large errors. Thus, some proper models, e.g., the orientation image model [22,25,26,27] and the piecewise polynomial model [20,25], were proposed to refine the EOPs of scanners and to produce high-precision topographic maps. However, the GPS/IMU is seldom integrated into the orbiters in satellite photogrammetry, e.g., Chang’E-1 and Chang’E-2.

Thirdly, in satellite photogrammetry, the data from both the push broom scanner and LAM are used for planet topographic reconstruction, e.g., Chang’E-1 [28]. Existing LAM data are usually integrated with the stereo CCD images to better recover the trajectory and calculate the topographic maps. Typical approaches [7,29,30] first utilized the existing LAM data to interpolate the 3D coordinates of the ground control points corresponding to the two extreme points on each scanline and then estimated the EOPs using the existing modeling of space resection. Later, a number of works [2,3,4,17,18,29,31,32] began with a rough estimation of the initial EOPs for each scanline using existing space resection modeling based on a very small number of ground control points directly selected from the existing LAM data and then integrated them into a rigorous adjustment framework for further refining the EOPs, co-registration between CCD images and LAM data and the topographic maps. Note that the bundle adjustment algorithm is easily influenced by the initial EOPs, and this also means accurate initial EOPs are important for the topographic reconstruction.

In this paper, we mainly focus on the lunar topographic reconstruction, and existing LAM data would be used to interpolate the altitude data of the ground control points. Hence, our concern is how to increase the error tolerance for the modeling of space resection, which is important for accurate EOPs’ estimation.

## 3. Specifications of Push Broom Scanners and Lunar LAM

### 3.1. Lunar Imagery with Push Broom Scanners

Recent lunar imagery with push broom scanners includes the three-line scanner on Chang’E-1 [1] and the two-line scanner on Chang’E-2 [2]. Their main scientific goal is to acquire stereo images of the lunar surface between 70S and 70N. As shown in Figure 1, they utilized a linear push-broom imagery technique to capture stereo CCD images in along-trip mode from different viewing directions. This also means that the images in different viewing directions could be acquired at the same time, while each scanline in the same CCD image has its own EOPs.

Table 1 compares the specifications of the scanners on Chang’E-1 and Chang’E-2, and two major differences can be summarized. One is that Chang’E-1 carried the three-line scanner, which captured three scanlines from the forward, nadir and backward viewing directions at each instant of time, while Chang’E-2 carried a two-line scanner, which captured two scanlines from the forward and backward viewing directions at each instant of time. Another major difference is that Chang’E-2 has a much higher spatial resolution for the CCD images. Things that are in common to both of them should be the lack of tracking data (equivalently the EOPs of scanners), and only the geographic (longitude and latitude) information of the first and last pixels for each scanline in the CCD image is provided.

### 3.2. Lunar LAM Data

Laser altimeter measurement (LAM) data are some of the important data sources for lunar topographic reconstruction. For each LAM point on the lunar surface, it records the 3D coordinates including the longitude, latitude and altitude information.

In recent years, several sets of LAM data have been collected with different spatial resolutions. For example, the LAM data collected from Chang’E-1 [6] contain more than nine million range measurements covering the entire lunar surface, with spatial resolutions of 1.4 km for the along-track direction and 7 km for the cross-track direction (at the Equator). The LAM data collected from Japanese SELENE [33] contain more than 10 million high-quality range measurements covering the entire lunar surface, with a height resolution of 5 m at a sampling interval smaller than 2 km, and hence, a global DEM with a spatial resolution of $0.5^{\circ }$ was produced. Besides, the LAM data collected from India’s Chandrayaan-1 [34] have a range of resolution better than 5 m. Moreover, the LAM data collected by the Lunar Orbiter Laser Altimeter (LOLA) onboard Lunar Reconnaissance Orbiter spacecraft (LRO) [35] have a spatial resolution of 10–12 m in the along-track direction and 1–2 km for the cross-track direction.

In our work, since we take the real data from Chang’E-1 as an example, we integrate the LAM data from Chang’E-1 to interpolate the 3D coordinates of the ground control points. However, it is noticeable that the LAM data are distributed unevenly and contain substantial noise (Figure 2).

## 4. System Overview

**Figure 3:**
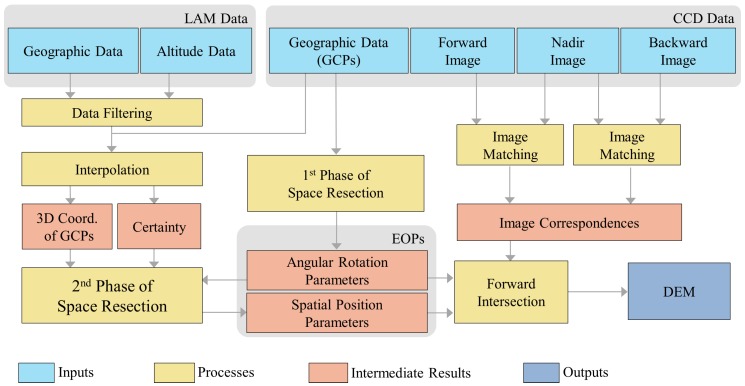
The framework of our system.

Figure 3 illustrates the framework of our system. The input of our system involves two types of data. One is the *stereo CCD images* of the lunar surface captured by the push broom scanner. In this paper, we take the three-line scanner as an example, which captures three sets of images corresponding to the forward, backward and nadir views. The *geographic data* of the first and last pixels for each scanline are also provided along with CCD images. In our experiment, we take the 3D points on the lunar surface corresponding to the first and last pixels for each scanline as the GCPs for recovering the trajectory of the orbiter. However, the recovery of trajectory relies on the complete 3D coordinates (longitude, latitude and altitude) of the GCPs, while their altitude data are lacking. Thus, another type of input data, the LAM data, which record the 3D coordinates of sample points on the lunar surface, are further utilized for interpolating the missing altitude data at the GCPs.

In our two-phase space resection approach, we first create a mathematical model that only involves the reliable geographic data of GCPs from CCD images to retrieve the angular rotation parameters of EOPs. Then, combing these estimated angular rotations with the complete 3D coordinates of the GCPs, as well as the computed certainty information, we deduce a close-form solution in the second phase of space resection to obtain the spatial position of the push broom scanner. Note that the certainty value is used to reflect the reliability of the interpolated altitude values. Lastly, the digital elevation model (DEM) can be synthesized using the forward intersection with the estimated EOPs and image matching results.

As mentioned, the altitude data at the GCPs are interpolated from the existing LAM data. However, the LAM data are distributed unevenly (Figure 2a) and contain noise (Figure 2b), which would inevitably bring different levels of errors to the interpolated altitude data. To eliminate these negative effects, we first perform data filtering in order to remove the incorrect LAM data using the sophisticated method proposed by Huang *et al*. [36]. Then, we employ Shepard’s method [37] to interpolate the altitude data for each GCP. To balance the selection of neighbors from different directions well, we modify their method by partitioning the surrounding region of each GCP into *K* uniform angle-spaced bins and select for the nearest neighbor evenly in each of the bins. Moreover, we further propose a novel metric to robustly measure the certainty of the interpolated altitude data at the GCPs. This enables us to selectively rely on the more accurate data than less accurate ones when computing the positional data of EOPs.

Please note that, in this paper, to achieve high precision in computation, instead of degree, we utilize radians as the units for longitude, latitude and angular rotation, indicated as “rad”. In addition, we utilize meters as the units for spatial position and altitude, indicated as “m”.

## 5. Remodeling of Space Resection

### 5.1. Motivation and Goal

The existing space resection model [5] estimates the EOPs by applying collinear equations, which are formulated based on the 3D coordinates of the GCPs. These collinear equations are nonlinear and complicated, and they consider each GCP with equal importance. The existing model has been proven to be effective in only the case if the data of the GCPs are reliable. Unfortunately, as we mentioned above, the altitude data of the GCPs in our work are interpolated from the existing LAM data, which usually contain errors. Based on the analysis of the mathematical model and experimental results, we can conclude with the following two issues when the existing model is applied in our application.

- The altitude data at the GCPs are error-prone, which inevitably affects subsequent procedures, including the EOPs’ estimation using the existing model of space resection and the topographic reconstruction using forward intersection [7]. This problem is clearly demonstrated using digital simulation data in Section 6. The experiments on the simulation data show that the estimation of angular rotations and spatial position is sensitive to the error of altitude data at the GCPs if using the existing model, and this brings serious errors to the reconstructed 3D coordinates.
- The existing model usually involves the use of an iterative process to determine the optimal solution using the nonlinear programming approach, which cannot guarantee obtaining a global optimal solution and is highly sensitive to the initial guess of the unknown parameters.

To tackle the above issues, we improve the approach based on an attractive finding about the angular rotation parameters of EOPs. Actually, these parameters can be estimated independent of the altitude data and only involving the geographic data at the GCPs. The main advantage here is the possibility to improve the estimation of EOPs in a divide-and-conquer manner. Thus, we can first estimate the angular rotation parameters based on the reliable geographic data at the GCPs, which were already available. Note that the reliability of the geographic data at the GCPs is the key to achieve the accurate estimated angular rotations. After that, with the accurate angular rotations, the collinear equations for space resection can be simplified into a linear optimization problem. Thus, a global optimal solution can always be achieved, and the initial values of unknown parameters have a minor effect on the final solution. Moreover, the computational cost for the optimization can also be reduced largely. As a result, both issues stated above are tackled gracefully at the same time.

In summary, our new modeling of space resection is performed in two phases. The first phase is to estimate the angular rotation parameters of EOPs based on the reliable geographic data (longitude and latitude information) at the GCPs. The second phase is to estimate the spatial position of EOPs by a linear optimization process aided with the estimated angular rotation parameters. In the following, we will introduce our two-phase modeling of space resection in detail.

### 5.2. Two-Phase Modeling of Space Resection

**Figure 4:**
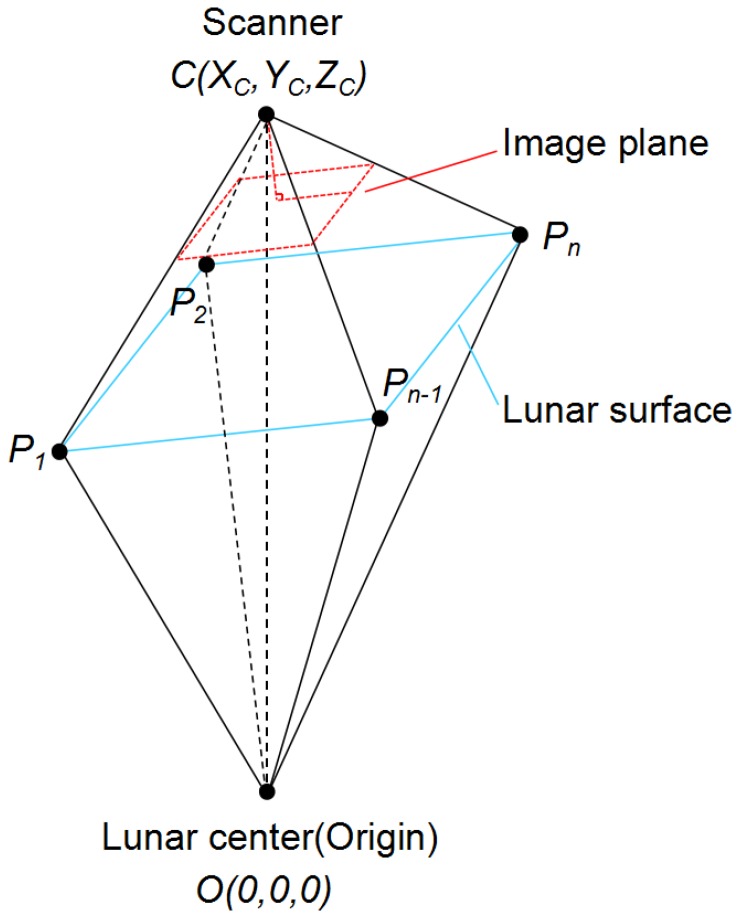
The coordinate system.

To better understand our two-phase model, we have to define the coordinate system (Figure 4). First, we use the lunar center as the origin in the 3D world coordinate system. The positive Z-axis is pointing towards the north pole of the Moon, and the positive X-axis is pointing towards the point on the Equator and longitude with 0. Then, we defined the spatial position of the push broom scanner as $C=(X_{C},Y_{C},Z_{C})$ and the *i*-th GCP on the lunar surface as $P_{i}$. The three angular rotation parameters of EOPs are defined as *θ*, ϕ, *ρ*, indicating the rotated angles along different axes in the world coordinate system. The rotational matrix *R* can then be represented in terms of the angular rotations as,

(1) $$R=\left[\begin{array}{ccc} a_{1} & a_{2} & a_{3}\\ b_{1} & b_{2} & b_{2}\\ c_{1} & c_{2} & c_{2} \end{array}\right]=\left[\begin{array}{ccc} 1 & 0 & 0\\ 0 & \cos(\theta ) & -\sin(\theta )\\ 0 & \sin(\theta ) & \cos(\theta ) \end{array}\right]\left[\begin{array}{ccc} \cos(\phi ) & 0 & -\sin(\phi )\\ 0 & 1 & 0\\ \sin(\phi ) & 0 & \cos(\phi ) \end{array}\right]\left[\begin{array}{ccc} \cos(\rho ) & -\sin(\rho ) & 0\\ \sin(\rho ) & \cos(\rho ) & 0\\ 0 & 0 & 1 \end{array}\right]$$

#### 5.2.1. Phase 1: Estimation of Exterior Angular Rotation

By observing the mechanism of push broom scanner (Figure 1 and Figure 4), we find that the angular rotation parameters can be determined only based on the geographic data and regardless of the altitude data at the GCPs. As illustrated in Figure 4, any GCP $P_{i}$ should form a plane $P_{i}OC$, and its normal vector $\vec{N_{i}}$ must be perpendicular to the direction $\vec{OC}$, which is determined by the lunar center *O* and the position of push broom scanner *C*. This can be presented by:

(2) $$\vec{N_{i}}\cdot \vec{OC}=0$$

Thus, by combing the equations from all GCPs, we can formulate our objective function as:

(3) $$\sum_{i=1}^{n}{\left(\vec{N_{i}}\cdot \vec{OC}\right)}^{2}=0$$

where *n* is the total number of GCPs at each instant of time. Note that the direction $\vec{OC}$ is shared for all GCPs, and the normal vector $\hat{N_{i}}$ can be obtained by the cross-product of two direction vectors $\vec{OP_{i}}$ and $\vec{CP_{i}}$, which is defined as:

(4) $$\vec{N_{i}}=\vec{OP_{i}}\times \vec{CP_{i}}$$

As for $\vec{OP_{i}}$, since the longitude $\varphi_{i}$ and latitude $\lambda_{i}$ of the GCP $P_{i}$ is known and *O* is the origin, this direction can be calculated by:

(5) $$\vec{OP_{i}}=\left[\begin{array}{c} cos\left(\varphi_{i}\right)\cdot cos\left(\lambda_{i}\right)\\ cos\left(\varphi_{i}\right)\cdot sin\left(\lambda_{i}\right)\\ sin(\varphi_{i}) \end{array}\right]$$

As for $\vec{CP_{i}}$, it can be calculated by transforming the corresponding pixel location $\left(px_{i},py_{i}\right)$ in image coordinates (2D) to world coordinates (3D), which can be presented by:

(6) $$\vec{CP_{i}}=R\left[\begin{array}{c} px_{i}\\ py_{i}\\ -f \end{array}\right]$$

where *f* is the focal length of the scanner. The values of *f* and $px_{i},py_{i}$ are all known. *R* is the rotational matrix defined in Equation (1) and determined by the exterior angular rotations at each instant of time. By substitution, we can deduce the objective function (Equation (3)) as:

(7) $$\sum_{i=1}^{n}{\left(\left[\begin{array}{c} cos\left(\varphi_{i}\right)\cdot cos\left(\lambda_{i}\right)\\ cos\left(\varphi_{i}\right)\cdot sin\left(\lambda_{i}\right)\\ sin(\varphi_{i}) \end{array}\right]\times \left(R\left[\begin{array}{c} px_{i}\\ py_{i}\\ -f \end{array}\right]\right)\cdot \vec{OC}\right)}^{2}=0$$

In this equation, the matrix *R* (determined by angular rotations *θ*, ϕ, *ρ*) and the direction $\vec{OC}$ are unknown, and our goal is to find the optimal matrix *R* that can minimize the objective function. To solve this non-linear optimization problem, we adopt a search-based method called the interior point method [38]. At each instant of time, we initialize the matrix *R* using the optimal *R* computed for the previous instant of time and limit the search range to $1.00\times 10^{-5}$, as the orbiter flies in a smooth trajectory most of the time. During this optimization process, the optimal matrix *R* and direction $\vec{OC}$ are all determined. However, we only need matrix *R* in the following procedure. Generally, at least four GCPs are needed to achieve the high accuracy for the DEM production as demonstrated in Table 2.

#### 5.2.2. Phase 2: Estimation of Exterior Spatial Position

With the accurate angular rotations, our goal is to simplify the collinear equations for space resection, so that the global optimal solution for camera position can always be achieved. According to the mechanism of the push broom scanner, there is an important constraint that the position of scanner *C*, GCP $P_{i}$ and the corresponding image pixel $S_{i}$ should lie on the same straight line. Thus, we can define a collinear equation:

(8) $$\frac{X_{CS_{i}}}{X_{CP_{i}}}=\frac{Y_{CS_{i}}}{Y_{CP_{i}}}=\frac{Z_{CS_{i}}}{Z_{CP_{i}}}$$

where $\left(X_{CP_{i}},Y_{CP_{i}},Z_{CP_{i}}\right)=\left(X_{C}-X_{P_{i}},Y_{C}-Y_{P_{i}},Z_{C}-Z_{P_{i}}\right)$, and the $\left(X_{CS_{i}},Y_{CS_{i}},Z_{CS_{i}}\right)$ can be calculated from the coordinate transformation:

(9) $$\left[\begin{array}{c} X_{CS_{i}}\\ Y_{CS_{i}}\\ Z_{CS_{i}} \end{array}\right]=R\left[\begin{array}{c} px_{i}\\ py_{i}\\ -f \end{array}\right]=\left[\begin{array}{ccc} a_{1} & a_{2} & a_{3}\\ b_{1} & b_{2} & b_{3}\\ c_{1} & c_{2} & c_{3} \end{array}\right]\left[\begin{array}{c} px_{i}\\ py_{i}\\ -f \end{array}\right]$$

Since *R* is a rotational matrix, $R^{T}=R^{-1}$, and Equation (9) can be further transformed to:

(10) $$\left[\begin{array}{c} px_{i}\\ py_{i}\\ -f \end{array}\right]=R^{T}\left[\begin{array}{c} X_{CS_{i}}\\ Y_{CS_{i}}\\ Z_{CS_{i}} \end{array}\right]$$

Based on Equations (8) and (10), two equations can be derived.

(11) $$\begin{array}{c} \frac{px_{i}}{-f}=\frac{a_{1}X_{CS_{i}}+b_{1}Y_{CS_{i}}+c_{1}Z_{CS_{i}}}{a_{3}X_{CS_{i}}+b_{3}Y_{CS_{i}}+c_{3}Z_{CS_{i}}}=\frac{a_{1}X_{CP_{i}}+b_{1}Y_{CP_{i}}+c_{1}Z_{CP_{i}}}{a_{3}X_{CP_{i}}+b_{3}Y_{CP_{i}}+c_{3}Z_{CP_{i}}}\\ \frac{py_{i}}{-f}=\frac{a_{2}X_{CS_{i}}+b_{2}Y_{CS_{i}}+c_{2}Z_{CS_{i}}}{a_{3}X_{CS_{i}}+b_{3}Y_{CS_{i}}+c_{3}Z_{CS_{i}}}=\frac{a_{2}X_{CP_{i}}+b_{2}Y_{CP_{i}}+c_{2}Z_{CP_{i}}}{a_{3}X_{CP_{i}}+b_{3}Y_{CP_{i}}+c_{3}Z_{CP_{i}}} \end{array}$$

Then, we replace $\left(X_{CP_{i}},Y_{CP_{i}},Z_{CP_{i}}\right)$ by $\left(X_{C}-X_{P_{i}},Y_{C}-Y_{P_{i}},Z_{C}-Z_{P_{i}}\right)$ in Equation (11) and deduce two collinear equations for each control point $P_{i}$ as the following.

(12) $$\begin{array}{cc} & \left[\begin{array}{ccc} px_{i}\cdot a_{3}+f\cdot a_{1} & px_{i}\cdot b_{3}+f\cdot b_{1} & px_{i}\cdot c_{3}+f\cdot c_{1}\\ py_{i}\cdot a_{3}+f\cdot a_{2} & py_{i}\cdot b_{3}+f\cdot b_{2} & py_{i}\cdot c_{3}+f\cdot c_{2} \end{array}\right]\left[\begin{array}{c} X_{C}\\ Y_{C}\\ Z_{C} \end{array}\right]\\ = & \left[\begin{array}{c} \left(px_{i}\cdot a_{3}+f\cdot a_{1}\right)\cdot X_{P_{i}}+\left(px_{i}\cdot b_{3}+f\cdot b_{1}\right)\cdot Y_{P_{i}}+\left(px_{i}\cdot c_{3}+f\cdot c_{1}\right)\cdot Z_{P_{i}}\\ \left(py_{i}\cdot a_{3}+f\cdot a_{2}\right)\cdot X_{P_{i}}+\left(py_{i}\cdot b_{3}+f\cdot b_{2}\right)\cdot Y_{P_{i}}+\left(py_{i}\cdot c_{3}+f\cdot c_{2}\right)\cdot Z_{P_{i}} \end{array}\right] \end{array}$$

Note that each GCP provides a pair of equations (Equation (12)). This implies that there are 2n equations in total when *n* GCPs are available. Since only three unknown variables $\left(X_{C},Y_{C},Z_{C}\right)$ need to be solved, ideally, two reliable GCPs (*i.e.*, four equations) are sufficient to solve this set of equations. Moreover, to reduce the effect of errors caused by LAM data interpolation, we further introduce a new term, the *certainty* of the interpolated altitude data at the GCP (defined in Section 5.3), as a weight in Equation (12), which enables our model to selectively rely on the more accurate data than less accurate ones. Hence, our objective function in the second phase can now be defined as:

(13) $$G_{min}=\sum_{i=1}^{n}\mu_{i}{\left(LM1_{i}\left[\begin{array}{c} X_{C}\\ Y_{C}\\ Z_{C} \end{array}\right]-RM1_{i}\right)}^{2}+\sum_{i=1}^{n}\mu_{i}{\left(LM2_{i}\left[\begin{array}{c} X_{C}\\ Y_{C}\\ Z_{C} \end{array}\right]-RM2_{i}\right)}^{2}$$

where:

$$LM1_{i}={\left[\begin{array}{c} px_{i}\cdot a_{3}+f\cdot a_{1}\\ px_{i}\cdot b_{3}+f\cdot b_{1}\\ px_{i}\cdot c_{3}+f\cdot c_{1} \end{array}\right]}^{T}\;LM2_{i}={\left[\begin{array}{c} py_{i}\cdot a_{3}+f\cdot a_{2}\\ py_{i}\cdot b_{3}+f\cdot b_{2}\\ py_{i}\cdot c_{3}+f\cdot c_{2} \end{array}\right]}^{T}$$

$$RM1_{i}=\left(px_{i}\cdot a_{3}+f\cdot a_{1}\right)\cdot X_{P_{i}}+\left(px_{i}\cdot b_{3}+f\cdot b_{1}\right)\cdot Y_{P_{i}}+\left(px_{i}\cdot c_{3}+f\cdot c_{1}\right)\cdot Z_{P_{i}}$$

$$RM2_{i}=\left(py_{i}\cdot a_{3}+f\cdot a_{2}\right)\cdot X_{P_{i}}+\left(py_{i}\cdot b_{3}+f\cdot b_{2}\right)\cdot Y_{P_{i}}+\left(py_{i}\cdot c_{3}+f\cdot c_{2}\right)\cdot Z_{P_{i}}$$

where $\mu_{i}$ is the certainty defined in Equation (16); more details of its definition will be covered in later sections. LM1,LM2,RM1,RM2 are the left side and the right side of the collinear equation (Equation (12)), respectively. To solve this function, since it has been simplified into a linear optimization problem, we can use a least square method to obtain its global optimal solution of $\left(X_{C},Y_{C},Z_{C}\right)$ for minimizing the energy $G_{min}$. Eventually, all parameters $\left(X_{C},Y_{C},Z_{C},\theta ,\phi ,\rho \right)$ in EOPs can be obtained through our two-phase estimation.

### 5.3. Certainty Determination for Interpolated Data

**Figure 5:**
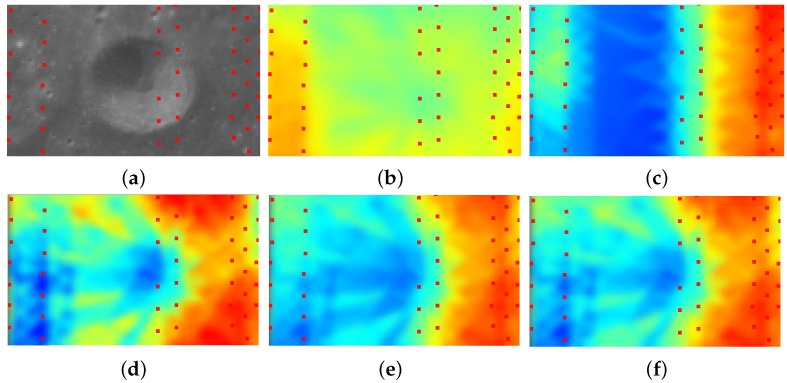
Certainty determination for interpolated data. (**a**) The CCD image containing a crater and LAM data (red dots) captured from Chang’E-1; (**b**) our interpolated altitude map; (**c**) the certainty map measured by the existing method (Equation (16)); (**d**) the cross-checking certainty map (Equation (15)); (**e**,**f**) the certainty maps measured by our certainty metric with α=0.5 and α=0.8, respectively. Note that for altitude maps, the red color indicates high altitude, while the blue color indicates low altitude. Similarly, for certainty maps, the red color indicates high certainty, while the blue color indicates low certainty. These rules are applicable for all figures in this paper.

As discussed above, the accuracy of the interpolated altitude data greatly affects the estimation of the spatial position of the scanner in the second phase of our space resection. However, the interpolated altitude data are not equally reliable due to the irregular distribution of LAM data. The LAM data usually fail to cover sufficient details (e.g., the crater boundary in Figure 5a). To avoid incorrectly adopting the unreliable interpolated data, we need to measure the certainty of the interpolated altitude data.

To measure the certainty of the interpolated data, the existing method [39] mainly focuses on the criteria of the distribution of surrounding points. More intuitively, if the surrounding points are closer to and more uniformly distributed around the interpolated point, this interpolated result should have high certainty. To simulate this situation, we evaluate the certainty based on the distribution of the surrounding points using the following equation.

(14) $$\begin{array}{c} \mu_{dist}\left(x\right)=\frac{\sum_{j=1}^{K}\text{max}\left(\left(D_{max}-d\left(x,x_{j}\right)\right),0\right)}{K\cdot D_{max}}.\\ d\left(x,x_{j}\right)=\text{arccos}\left(\cos\left(\varphi \right)\cdot \cos\left(\varphi_{j}\right)\cdot \cos\left(\lambda -\lambda_{j}\right)+\sin\left(\varphi \right)\cdot \sin\left(\varphi_{j}\right)\right) \end{array}$$

where $d(x,x_{j})$ is the great-circle distance between two points. *x* indicates the interpolated point; $x_{j}$ indicates the *j*-th nearest LAM point; and *K* is the total number of nearest point; φ(λ) and $\varphi_{j}\left(\lambda_{j}\right)$ are the latitude (longitude) for *x* and $x_{j}$, respectively. Note that, for the LAM data captured from Chang’E-1, generally, the angular distance of two LAM points is less than $D_{max}=7/1700$ under the units of “rad” (refer to Section 3.2).

Figure 5c illustrates the certainty map using Equation (14), in which the regions with large errors (e.g., crater) are incorrectly determined to have similar certainty values as other regions, due to their similarities in the distribution of the surrounding LAM points. However, in fact, similar distributions may not always lead to similar certainty values, since complicated geometries (e.g., craters) usually require dense sampling to produce accurate interpolated results. Unfortunately, in our work, the geometry of the lunar surface, as well as the relationship between the complexity of the geometry and the density of the sampling points are both unavailable.

To estimate certainty robustly, our idea is to cross-check the available altitude samples in the LAM data to their corresponding interpolated values. Specifically, at the location of a LAM sample, we first calculate its interpolated altitude result $h^{\prime }$. Then, the certainty is evaluated using Equation (15) by comparing to its real altitude value *h*. As a result, the certainty values at all sampled LAM locations can be obtained, we then fill up the whole certainty map by interpolation with a similar scheme to altitude interpolation and illustrate the result in Figure 5d. Note that the certainty values on the LAM points actually have already revealed the relationship between the complexity of geometry and the density of the sampling points to some extent.

(15) $$\mu_{cross}\left(x\right)=\frac{\max\left(E_{max}-\right|h\left(x\right)-h^{{}^{\prime }}\left(x\right)\left|,0\right)}{E_{max}}$$

where $E_{max}$ denotes the largest error, which is set to 2000 m in our work. This is because most of the altitude values on the lunar surface are less than 2000 m.

Finally, we take the above two proposed aspects into account and define our certainty metric as:

(16) $$\mu \left(x\right)=\alpha \cdot \mu_{dist}\left(x\right)+\left(1-\alpha \right)\cdot \mu_{cross}\left(x\right)$$

where *α* is used to balance the importance of the two aspects. Figure 5e,f shows our certainty maps when α=0.5 and α=0.8, respectively, and α=0.5 is usually used in our work. It is observed that our certainty map is much more reasonable compared to Figure 5c produced by the existing method. Furthermore, our result is more consistent in various regions, especially where a sharp change of altitude exists (e.g., crater). We can clearly observe that places with large interpolation errors are assigned low certainty values.

## 6. Digital Simulation

A complete and reliable evaluation is required to evidence that our model is superior to the existing model. However, due to the lack of ground truth of lunar terrain, we cannot conduct an evaluation objectively. Instead, we performed a simulation for validating our two-phase space resection. We generate a simulated lunar surface, simulated LAM data, simulated trajectories of the orbiter and simulated stereo images. The settings for the simulated data are similar to the real situations of Chang’E-1 and Chang’E-2. We then conducted three validations based on the simulated data, with respect to the *effectiveness*, *robustness* and *contribution to topographic reconstruction*, respectively.

### 6.1. Simulated Lunar Surface

**Figure 6:**
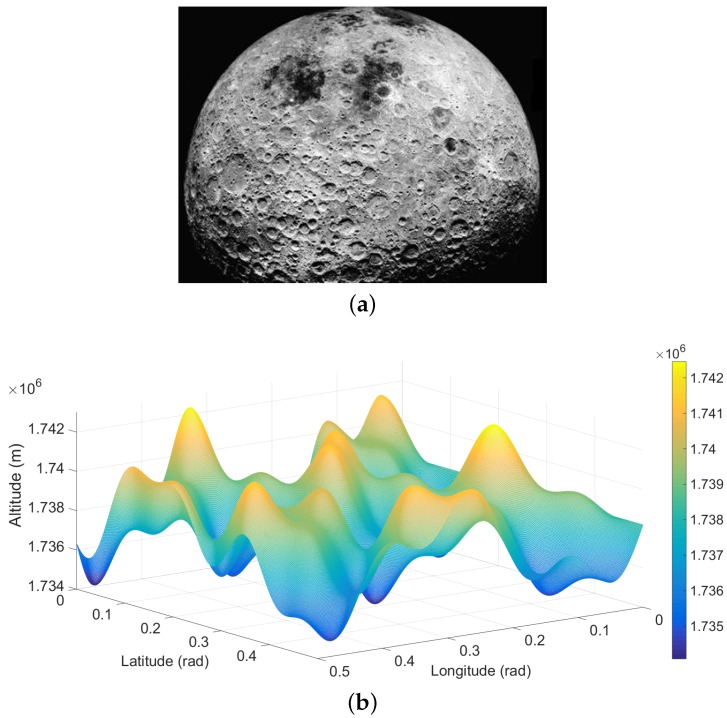
(**a**) The photo captured from the lunar surface; (**b**) our simulated lunar surface.

Obviously, there are many craters on the lunar surface (Figure 6a). These craters appear on the surface randomly, and their boundaries change sharply in low scale, but smoothly in high scale. To simulate this natural surface, we designed a continuous terrain function $\tilde{H}$ composed of multiple wavelets. This function represents the height for points with longitude *λ* and latitude φ, which is:

(17) $$\begin{array}{c} \tilde{H}\left(\lambda ,\varphi \right)=1,738,200+\left(200\varphi +9000\right)\sin\left(r_{1}\lambda \right)\cos\left(r_{2}\varphi \right)+\left(200*2\pi \lambda +9000\right)\sin\left(r_{3}\lambda \right)\cos\left(r_{4}\varphi \right) \end{array}$$

where the constants 1,738,200 and 9000 are the radius of the reference lunar surface and the maximum altitude variance on the real lunar surface, respectively. They are used to represent the altitude magnitude of the simulated surface. To introduce random effects, we integrate the term of 200φ to change the magnitude slightly with the change of latitude and $r_{i}$ to generate a wavelet with a random period.

In our simulation, we set $0<\varphi <\frac{\pi }{2}$ and 0<λ<2π for simulating the hemisphere of the Moon. Figure 6b shows an example region of the generated surface using our terrain function with the setting of $r_{1}=40,r_{2}=30,r_{3}=15,r_{4}=20$. This generated surface has a similar appearance to the real lunar terrain.

### 6.2. Simulated Orbiter Trajectory

In our simulation, the next step is to generate the trajectory of the orbiter. According to the specifications in Table 1, the orbiters of Chang’E-1 and Chang’E-2 flew around the Moon at distances of 200 ± 2 km and 100 ± 1 km from the reference surface of the Moon, respectively. The trajectory for one circular orbit is circle-like shaped, and the scanner on the orbiter should always point to the center of the Moon with error ±0.0523 due to unavoidable situations (e.g., vibration or gravity).

To follow this real case, we simulate the trajectory of a circular orbit by the time-dependent wavelets. Hence, the position of simulated scanner $\left({\tilde{X}}_{C}\left(t\right),{\tilde{Y}}_{C}\left(t\right),{\tilde{Z}}_{C}\left(t\right)\right)$ and its orientation $\left(\tilde{\theta }\left(t\right),\tilde{\phi }\left(t\right),\tilde{\rho }\left(t\right)\right)$ at time *t* are defined as:

(18) $$\begin{array}{ccc} \tilde{\theta }\left(t\right) & = & \pi +\frac{2\pi }{T}t+0.0523\sin\left(r_{1}t+r_{1}^{\prime }\right)\\ \tilde{\phi }\left(t\right) & = & 0.0523\sin\left(r_{2}t+r_{2}^{\prime }\right)\\ \tilde{\rho }\left(t\right) & = & 0.0523\cos\left(r_{3}t+r_{3}^{\prime }\right)\\ {\tilde{X}}_{C}\left(t\right) & = & v\sin(r_{4}t+r_{4}^{\prime })\\ {\tilde{Y}}_{C}\left(t\right) & = & \left(H+v\sin\left(r_{5}t+r_{5}^{\prime }\right)\right)\sin\left(\tilde{\rho }\left(t\right)\right)\\ {\tilde{Z}}_{C}\left(t\right) & = & \left(H+v\cos\left(r_{6}t+r_{6}^{\prime }\right)\right)\cos\left(\tilde{\rho }\left(t\right)\right) \end{array}$$

where 0≤t≤T, *T* controls the stopping time of simulation. *H* is the height of the simulated circle orbit, and *v* is the variance. They are set as 200,000 and 2000 for Chang’E-1, respectively. For Chang’E-2, *H* and *v* are set as 100,000 and 1000, respectively. $r_{i}$ and $r_{i}^{\prime }$ are random variables to control the period and the offset of the wavelets, which is used to simulate the random small fluctuation of the trajectory.

### 6.3. Simulated LAM Data

**Figure 7:**
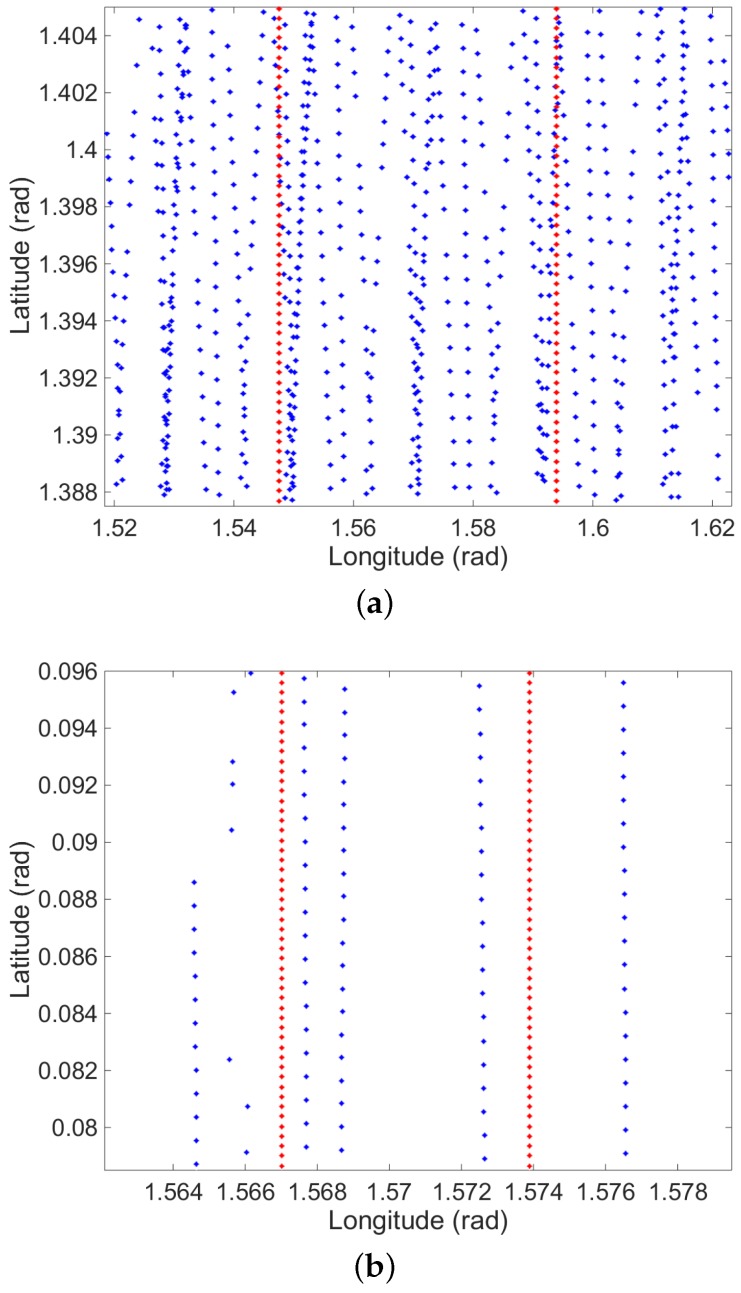
The distribution of simulated LAM data close to the pole (**a**) and Equator (**b**). The X and Y axes correspond to longitude and latitude, respectively. The blue dots indicate the simulated LAM data, and the red dots indicate the simulated GCPs for Chang’E-1.

In our simulation, we directly follow the distribution of LAM data from Chang’E-1. Note that Chang’E-2 does not provide the LAM data. Specifically, we substitute the longitude and latitude values of the existing LAM data from Chang’E-1 and generate new altitude values by using Equation (17). These new values form our simulated LAM data. Figure 7 shows the distribution of the simulated LAM data (blue dots) on the pole at about 0.1396 latitude and on the Equator at about 0.0872 latitude, respectively. To correctly visualize the spatial density of the simulated LAM data, these two figures almost cover the same size of spatial areas. Note that the simulated LAM data are sampled with higher density on the region near the pole, compared to the region near the Equator. This verifies the consistency between the simulated LAM data and the real LAM data from Chang’E-1.

### 6.4. Simulated Stereo Images

In our simulation, we prepare two sets of stereo images to simulate the CCD images from Chang’E-1 and Chang’E-2. Firstly, to simulate the images from Chang’E-1, we need to generate three images according to three different views (*i.e.*, forward, nadir and backward), in which each pixel records the 3D coordinates of its corresponding location on the simulated lunar surface. Thus, the matched pixels among different viewing images can be determined by finding the pixels containing the same 3D coordinates. Actually, this is exactly what CCD images contribute for topographic reconstruction. Hence, we can substitute the simulated stereo images for the CCD images captured by Chang’E-1. Furthermore, we also simulate the images from Chang’E-2 by generating two images according to two different views (*i.e.*, forward and backward).

To generate these simulated images, we need to calculate the 3D coordinates of the corresponding location on the simulated lunar surface for every pixel. Due to the optical characteristic, the ray from the scanner to a certain pixel must also pass through its corresponding location *P* on the simulated lunar surface. Thus, the 3D coordinates $\left({\tilde{X}}_{P},{\tilde{Y}}_{P},{\tilde{Z}}_{P}\right)$ can be determined by the ray-point intersection, which is:

(19) $${\left[\begin{array}{c} {\tilde{X}}_{P}\\ {\tilde{Y}}_{P}\\ {\tilde{Z}}_{P} \end{array}\right]}^{T}={\left[\begin{array}{c} {\tilde{X}}_{C}\\ {\tilde{Y}}_{C}\\ {\tilde{Z}}_{C} \end{array}\right]}^{T}+l\cdot {\tilde{S}}_{ij}\cdot R$$

where ${\tilde{X}}_{C},{\tilde{Y}}_{C},{\tilde{Z}}_{C}$ are the spatial position coordinates of the scanner and *R* is the rotation matrix defined in Equation (1). *l* is the length of the ray to intersect with the corresponding location on the simulated lunar surface. ${\tilde{S}}_{ij}$ is the 2D coordinates for each pixel in the captured image, which is:

(20) S˜i,j=X˜Si,j,Y˜Si,j,f==ΔXi,ΔY·(j-YY,f

where *i* indicates the index of the viewing directions and *j* indicates the pixel index in each scanline. For Chang’E-1, i=1,2,3 corresponds to backward, nadir and forward view, respectively; ΔX={-6.9993,0,6.9993}; j=1,⋯,512; ΔY=3.5729; Y=255.5; f=23.33. For Chang’E-2, i=1,2 corresponds to backward and forward view, respectively; ΔX={-44.6683,20.2800}; j=1,⋯,6144; ΔY=55.3916; Y=3071.5; f=144.3. Thus, ${\tilde{S}}_{i,j}$ can be determined in advanced, and only *l* on the right-hand side of Equation (19) is unknown.

To obtain the intersection point between the ray from the scanner to a certain pixel and the simulated lunar surface, we integrate their corresponding equations (Equations (17) and (19)) and solve this problem using an iterative process [40]. Hence, the corresponding 3D coordinates $\left({\tilde{X}}_{P},{\tilde{Y}}_{P},{\tilde{Z}}_{P}\right)$ for each pixel in the simulated images can be obtained.

Based on the simulated stereo images, we can easily obtain the simulated GCPs, which are the corresponding locations on the simulated lunar surface for the first and last pixels on each line of the simulated images.

### 6.5. Validation

Based on the simulated data, we conduct three different validations. The first validation is to test the effectiveness of our model based on the error-free data. The second validation is to test the robustness of our model based on the noisy data. The third validation is to test the contribution of our model to topographic reconstruction based on the noisy data. We carry out the first two validations only based on the simulated data for Chang’E-1, since the three-line scanner carried by Chang’E-1 provided more GCPs (six in total), which are more valuable for comparison. However, we test the contribution to topographic reconstruction based on the simulated data both for Chang’E-1 and Chang’E-2, since these two datasets have different resolutions of their simulated images which leads to different precisions of image matching results and, finally, affects the topographic reconstruction.

For better illustration, we indicate the existing space resection method as “exist.”, our model without the certainty term as “ours-1”, and our model with the certainty term as “ours-2”.

#### 6.5.1. Effectiveness

To validate the effectiveness of our two-phase modeling of space resection, we directly use the error-free data of the simulated GCPs to estimate the EOPs for avoiding the influence of errors. We test the influence of the number of GCPs for the EOPs estimation. The last two rows in Table 2 compare the errors of the estimated angular rotation and spatial position using different numbers of control points. It demonstrates that, generally, the four GCPs are sufficient to achieve the high accuracy of estimated EOPs, which can guarantee producing precise topographic maps (referring to Table 4). Table 2 also shows the comparison between our results with those by the existing model. Obviously, both the existing model and our model can achieve high accuracy based on the error-free data when the number of GCPs is equal to or larger than four. Note that we only show “ours-1” in this validation. This is because the altitude data of GCPs are generated by simulation instead of interpolating from sparse data. Thus, there is no issue of certainty, and “ours-2” should produce the same results as “ours-1” in this validation.

#### 6.5.2. Robustness

**Figure 8:**
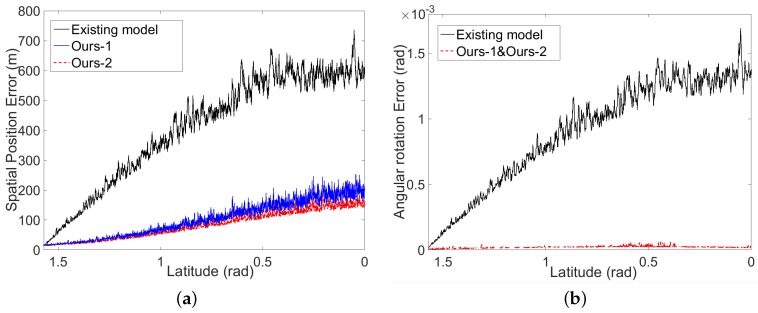
Errors of the estimated EOPs covering the trajectory from the pole (π/2 latitude) to the Equator (0 latitude). (**a**) The errors of the estimated spatial position; (**b**) the errors of the estimated angular rotation. The red and black curves indicate the results by our model and the existing model, respectively.

To validate the robustness of our model, we introduce the errors to the data of GCPs in two ways. First, we add some small perturbations to the simulated altitude data at the GCPs and estimate the EOPs with these noisy data. Table 3 shows the errors of estimated EOPs when different levels of error are added. Thanks to our two-phase model of space resection, the angular rotation estimation is independent of the altitude data. Hence, the errors of angular rotation of our model (the third and fifth rows) are always small. However, for the existing model, both angular rotation estimation and spatial position estimation are easily affected by the errors of the altitude data. Secondly, we add errors to the simulated GCPs by mimicking the data collection of Chang’E-1. That is to say that only the longitude and latitude data at the GCPs are provided, and the altitude data at the GCPs are obtained by our interpolation scheme from the simulated LAM data. Based on these noisy data, we first estimate the EOPs using our model and the existing model, respectively, and further calculate the errors of the estimated EOPs by comparing to the simulated real EOPs. As illustrated in Figure 8, the errors of estimated EOPs by our model (blue curves for ours-1 and red curves for ours-2) and the existing model (black curves) are both increased along the direction from the pole to the Equator. That is because the simulated LAM data (Figure 7) are distributed more sparsely when they are closer to the Equator, and hence, large errors would be introduced to the interpolated altitude data closer to the equator. However, thanks to our two-phase model, the error of angular rotation is stable and small, and the error of spatial position is also improved largely, since our model can guarantee obtaining global the optimal solution, compared to the results of the existing model. It is also clear that integrating the certainty term can further improve the accuracy of the spatial position. Recall that the angular rotation is estimated in the first phase of our space resection before the interpolation of LAM data. Thus, the angular rotation will not be affected by the certainty term. This is why we only show “ours-1” in Figure 8b.

#### 6.5.3. Contribution to Topographic Reconstruction

Our final objective is to increase the accuracy of the reconstructed lunar topography based on the provided data. Hence, we validate the contribution of our model to the topographic reconstruction of the Moon. Generally, the lunar topography is reconstructed using forward intersection [7] based on the EOPs data and the corresponding pixels among different viewing images. This implies that the accuracy of the reconstructed results largely relates to the reliability of the EOP data and the corresponding pixels. To test the robustness, on the one hand, we introduce errors into the EOP data to create synthetic testing data. On the other hand, we utilize the two simulated datasets for Chang’E-1 and Chang’E-2 to represent two levels of reliability for the corresponding pixels, since these two datasets have different resolutions of the simulated images and, hence, produce different reliability (uniformity and density) of the corresponding pixels.

**Figure 9:**
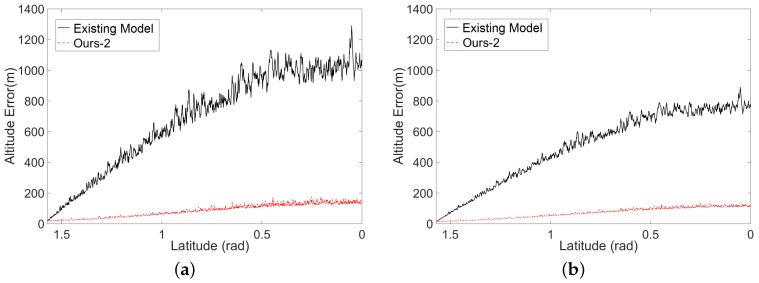
Errors of the reconstructed altitude values for pixels on the simulated images covering the areas from the pole (π/2 latitude) to the Equator (0 latitude). The errors of the reconstructed altitude values on the same latitude are averaged. The red and black curves indicate the reconstructed error based on our estimated EOPs and the estimated EOPs by the existing model, respectively. (**a**) The reconstructed errors based on the simulated data for Chang’E-1; (**b**) the reconstructed errors based on the simulated data for Chang’E-2.

To introduce the errors to the EOPs data, we also do this in two ways. One is to directly add different levels of errors to the actual angular rotation and spatial position individually. Table 4 shows the reconstructed errors according to different levels of error in angular rotation and spatial position. It is noticeable that the errors of the reconstructed altitudes increase to hundreds of meters, which is almost unacceptable when the error of angular rotation is larger than $1.0e^{-4}$ rad or the error of spatial position is larger than $1.0e^{2}$ m. At the same time, the reconstructed error for Chang’E-2 is smaller, since the simulated data for Chang’E-2 can produce a larger amount of corresponding points based on the simulated stereo images, compared to the simulated data for Chang’E-1. Another way is to estimate the EOPs based on the interpolated altitude data at the simulated GCPs. Then the altitude value for each pixel in the simulated images is reconstructed based on the estimated EOPs. Figure 9 shows that the errors of the reconstructed altitude values have been largely suppressed based on our estimated EOPs, comparing to the results based on the estimated EOPs by the existing model of space resection. In addition, the simulated data of Chang’E-2 now have higher precision reconstructed altitude values due to the higher reliability of the corresponding points among different viewing images.

## 7. Experiment on Real Data

Experiments on real data were also conducted for evaluation. However, in this paper, we only focus on the real data from Chang’E-1, since we had not obtained the whole set of Chang’E-2 data. Moreover, there is no LAM data in Chang’E-2; the LAM data in Chang’E-1 are still required even if we use the Chang’E-2 data. Based on the data from Chang’E-1, we carry out two comparisons: (1) errors of the estimated EOPs; and (2) errors of the reconstructed altitude maps for the CCD images. Note that in this section, our model indicates the second case of our model considering the certainty term and using our interpolation scheme. Moreover, unlike the simulation, we cannot directly calculate the stereo correspondence for imagery data. Thus, we further propose a SIFT-guided and normalized cross-correlation (NCC)-based image matching method for obtaining accurate matching results.

### 7.1. Stereo Image Matching

**Figure 10:**
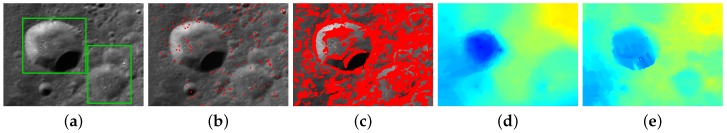
Image matching using SIFT and SIFT + normalized cross-correlation (NCC). (**a**) The original CCD images; (**b**,**c**) the features (red dots) detected by SIFT and SIFT + NCC, respectively; (**d**,**e**) the reconstructed altitude maps based on (**b**) and (**c**), respectively.

The accuracy of image matching is important for topographic reconstruction using forward intersection. One traditional way is to perform the feature-based matching methods, such as SIFT [41] (Figure 10b). Unfortunately, dense corresponding points are difficult to acquire due to the insufficient features of the lunar surface. Directly reconstructing the altitude map (Figure 10d) based on these sparse features is inadequate to recover the complete 3D structures. Some important objects may not be clearly represented or even disappear in this altitude map (e.g., the craters in the green frames of Figure 10a). In addition, the patch-based matching methods for each pixel are commonly used to obtain the dense corresponding points, such as the NCC [42]. However, they are usually computationally expensive and require many tedious manually tunings of parameters. To obtain the dense corresponding points among the stereo CCD images with high efficiency, we propose a SIFT-guided and NCC-based image matching method.

We first extract the SIFT features from the stereo images and compute the deviations of these correspondences. For the pixels without correspondence, due to the smoothness assumption of the surface, their transformation should be more or less similar to the nearest pixels, which have SIFT correspondences. This implies that the deviations of the pixels without correspondences can be interpolated using our interpolation scheme based on the known deviations in their neighborhood, and thus, we can use the interpolated deviation as the initial guess to find their corresponding pixels. After that, we refine these correspondences by searching in a bounded region and maximizing the normalized cross-correlation score. Figure 10c shows the pixels with correspondences using our matching method. The thresholds for SIFT and NCC (with a 5×5 kernel size) are experimentally set as 150 and 0.93, respectively. In our experiments, our matching method can increase the number of reasonable correspondences by 500 times. It is also noticeable that missing objects in Figure 10d become sharper and identifiable in Figure 10e. This verifies that our matching method can preserve more details for the topographic reconstruction.

### 7.2. Comparison with the Existing Space Resection Model

We perform two comparisons with the existing model for space resection. Firstly, we compare the estimated EOPs, which are directly produced by our model and the existing model. Furthermore, to validate the contribution of our model for the topographic reconstruction, we also compare the reconstructed altitude maps for CCD images based on the estimated EOPs by our model and the existing model.

**Figure 11:**
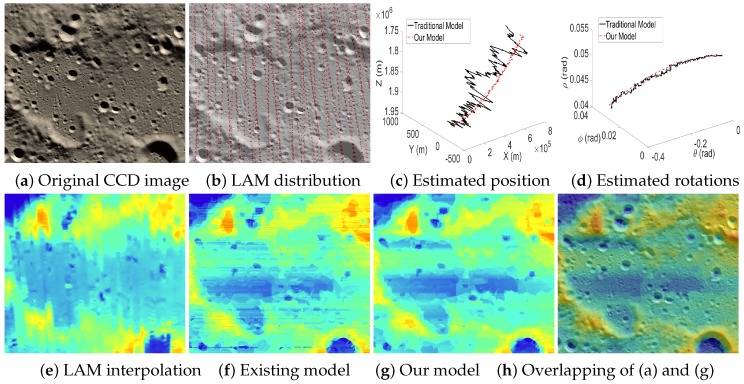
Case 1 with dense LAM distribution. (**c**,**d**) The curves of the spatial position and angular rotations estimated by the existing model (black) and our model (red); (**f**,**g**) the reconstructed altitude maps for (**a**) based on the estimated EOPs by the existing model and our model, respectively.

**Figure 12:**
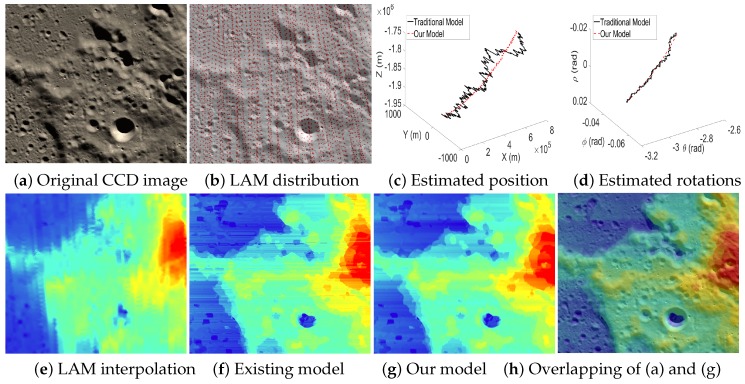
Case 2 with a dense LAM distribution.

Figure 11 and Figure 12 visually compare the results when the test cases have dense LAM distributions. This guarantees the reliability of the interpolated data at the GCPs and, hence, produces accurate EOPs. Note that the estimated EOPs (c–d) by our model (red curves) and existing model (black curves) are similar, and the reconstructed altitude maps based on the estimated EOPs by our model (g) and the existing model (f) are also similar. However, it is still observed that compared to the map by LAM interpolation (e), our altitude map (g) contains more details (e.g., the boundaries of craters are more consistent with the textures in CCD images). Moreover, the reconstructed altitude map (f) contains the artifacts of unreasonably horizontal lines since the EOPs estimated by the existing model contain big errors. However, our model can suppress the big errors of the estimated EOPs and, hence, produces a more reasonable altitude map (g).

**Figure 13:**
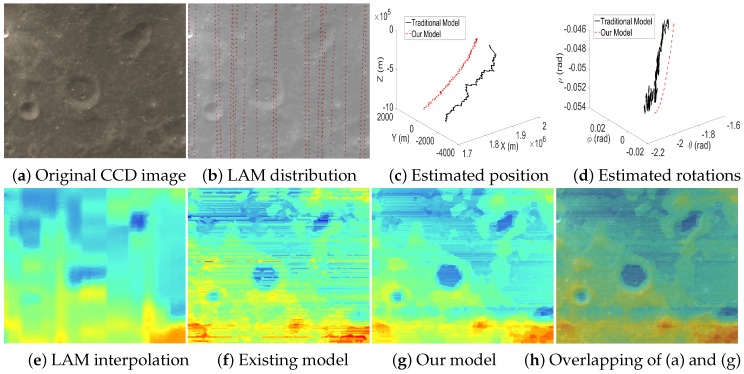
Case 3 with a sparse LAM distribution.

**Figure 14:**
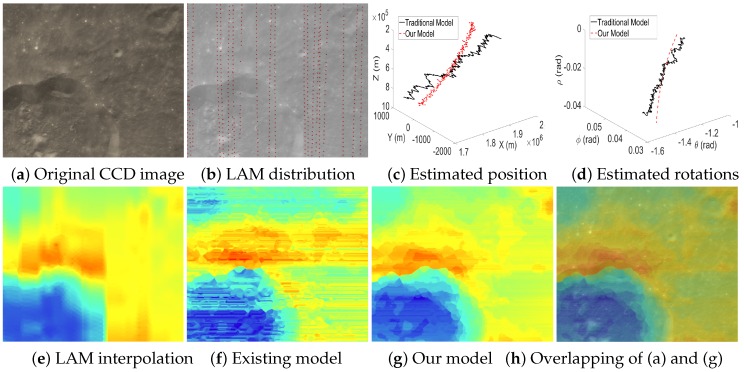
Case 4 with a sparse LAM distribution.

**Figure 15:**
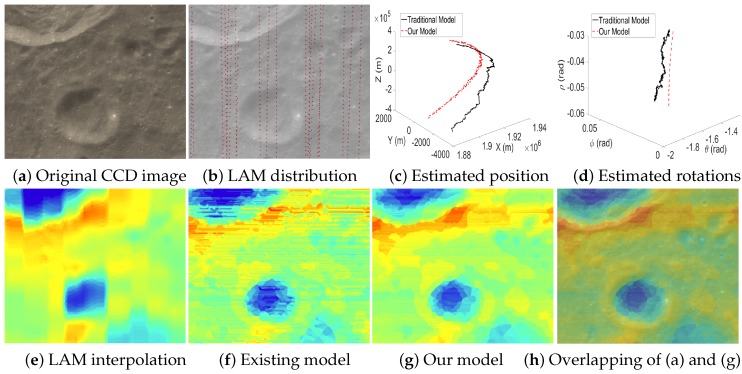
Case 5 with a sparse LAM distribution.

Figure 13, Figure 14 and Figure 15 are the test cases located near the Equator of the Moon. The distribution of LAM data in these areas is relatively sparse, which leads to big errors in the interpolated altitude data. Therefore, it is clear that the estimated EOPs (c–d) by our model and the existing model are quite different. Moreover, in the reconstructed altitude map (f) derived from the existing model, the artifacts of unreasonably horizontal lines are even serious, and many details are also lost. Thanks to our two-phase model, we can obtain an accurate angular rotation and spatial position even if a part of the altitude data contains big errors, and hence, our reconstructed altitude map (g) can recover the details of the terrain well.

### 7.3. Time Performance

All of our experiments ran on a PC with an Intel i7 CPU, 16 GB RAM. Table 5 shows the time performance of our model and the traditional model of space resection. It is obvious that our model is much more efficient than the traditional one. For image matching, our enhanced matching takes a bit longer than the case of only applying SIFT, while it is greatly faster than solely applying NCC.

## 8. Conclusions

In this paper, we propose a novel two-phase modeling of space resection that can be used to estimate accurate EOPs. It is motivated by an interesting finding that for the mechanism of the push broom scanner, the estimation of angular rotations of EOPs can be independent of the altitude data, and only involving the longitude and latitude data at the GCPs. Hence, we divide the modeling of space resection into two phases. Firstly, we estimate the angular rotation based on the reliable longitude and latitude data provided along with CCD images using our proposed mathematical model. Then, with the accurate angular rotations, we simplify the collinear equations of space resection into a linear optimization problem, and hence, the global optimal spatial position of EOPs can always be achieved in the second phase. Finally, our experiments on the simulated data and real data further validate the effectiveness and robustness of our model on the EOPs’ estimation, as well as the contribution to the topographic reconstruction.

Since the accurate estimated EOPs by our method can be used as an initial value for bundle adjustment, we manage to integrate our model into the bundle adjustment in the future for obtaining accurate DEM. Secondly, our model currently only accepts the GCPs at the first and last pixels in each scanline, while they may not always be reliable. In the future, we will extend our model to integrate more GCPs on any location in the scanlines, which should be able to significantly improve the accuracy of the estimated EOPs. Moreover, although our model can reduce the steaks in the reconstructed altitude maps largely compared to the existing space resection model, some streaks still remained in our model. To overcome this problem, we will develop a new smoothing scheme on our recovered trajectory, which can prevent spurious variations, while catching the real oscillations of the camera in angular orientation over time.

## Figures and Tables

**Table 1:** The specifications of push broom scanners on Chang’E-1 and Chang’E-2.

	Chang’E-1	Chang’E-2
Launched year	2007	2010
Altitude	200 km ±2 km	100 km/15 km ±1 km
Scanner type	Three-line scanner	Two-line scanner
Focal length	23.33 mm	144.3 mm
FOV	$40^{\circ }$	$42^{\circ }$
Camera	One CCD camera	Two single-line CCD arrays
CCD size	14.34 mm	62.06 mm
Pixel size	14 μm	10.1 μm
Spatial resolution	120 m	7 m (100 km altitude)
		1.3 m (15 km altitude)
Resolution	1024 × 1024	6144 × 1 × 2
Scan line	11th, 512th, 1013th of CCD	1st, 2nd
View Angle	$16.7^{\circ }$ (forward)	$8^{\circ }$ (forward)
	$0^{\circ }$ (nadir)	$-17.2^{\circ }$ (backward)
	$-16.7^{\circ }$ (backward)	

**Table 2:** Error of estimated Exterior orientation parameters’ (EOPs) with different numbers of GCPs based on error-free data. Here, “GCP Num.” indicates the number of ground control points; “Ang. Err.” indicates the error of angular rotation; “Pos. Err.” indicates the error of spatial position. When the number of GCPs is ≥3, all GCPs should not lie in a single straight line.

	GCP Num. 2	GCP Num. 3	GCP Num. 4	GCP Num. 5	GCP Num. 6
Ang. Err. by exist. (rad)	$9.90\times 10^{-3}$	$1.51\times 10^{-4}$	$5.55\times 10^{-5}$	$3.46\times 10^{-5}$	$8.40\times 10^{-6}$
Pos. Err. by exist. (m)	4604.97	69.92	26.11	16.70	3.75
Ang. Err. by ours-1 (rad)	$4.57\times 10^{-2}$	$1.20\times 10^{-4}$	$4.13\times 10^{-5}$	$2.83\times 10^{-5}$	$2.09\times 10^{-5}$
Pos. Err. by ours-1 (m)	11710.52	56.57	18.70	13.13	9.76

**Table 3:** Error of estimated EOPs by adding different perturbations to real altitude values at the GCPs. Here “Alti. Err.” indicates the error of altitude values.

	Alti. Err. 1000 m	Alti. Err. 300 m	Alti. Err. 100 m	Alti. Err. 30 m	Alti. Err. 0 m
Ang. Err. by exist. (rad)	$3.56\times 10^{-3}$	$9.16\times 10^{-4}$	$4.03\times 10^{-4}$	$1.88\times 10^{-4}$	$8.40\times 10^{-6}$
Pos. Err. by exist. (m)	1639.26	423.31	188.29	88.11	3.75
Ang. Err. by ours-1 (rad)	$2.05\times 10^{-5}$	$2.15\times 10^{-5}$	$2.03\times 10^{-5}$	$2.03\times 10^{-5}$	$2.09\times 10^{-5}$
Pos. Err. by ours-1 (m)	577.37	174.25	61.74	22.31	8.93
Ang. Err. by ours-2 (rad)	$2.05\times 10^{-5}$	$2.15\times 10^{-5}$	$2.03\times 10^{-5}$	$2.03\times 10^{-5}$	$2.09\times 10^{-5}$
Pos. Err. by ours-2 (m)	512.61	160.49	54.37	20.85	9.76

**Table 4:** The error of the reconstructed altitude data using forward intersection and based on the EOPs containing different levels of errors. Here, “Recon. Err. -1" and “Recon. Err. -2" indicate the errors of reconstructed altitude data based on the simulated data for Chang’E-1 and Chang’E-2 respectively.

	Ang. Err. (rad)			Pos. Err. (m)		
	$1.00\times 10^{-5}$	$l.00\times 10^{-4}$	$l.00\times 10^{-3}$	$l.00\times 10^{1}$	$l.00\times 10^{2}$	$l.00\times 10^{3}$
Recon. Err. -1 (m)	13.72	67.26	472.80	1.82	119.22	904.31
Recon. Err. -2 (m)	12.20	51.74	341.26	1.53	81.22	638.41

**Table 5:** Time performance of our model and the existing model of space resection.

TLS Image Resolution	1000×512	2000×512	5000×512
Existing model	74.8 s	140.6 s	328.1 s
Our model	29.9 s	56.1 s	120.3 s
SIFT	5.0 s	10.0 s	23.2 s
NCC	>1 h	>3 h	>6 h
SIFT + NCC	84.7 s	167.3 s	320.0 s
